# Heart failure reporting signals associated with concomitant mavacamten and CYP inhibitors in obstructive hypertrophic cardiomyopathy: a real-world pharmacovigilance study with propensity score matching

**DOI:** 10.3389/fphar.2026.1846852

**Published:** 2026-07-21

**Authors:** Daqiu Chen, Yanqing Wu, Zhanxiong Xie, Wenchao Qiu, Xiaomai Huang, Shanghua Xu, Shunxiang Luo

**Affiliations:** Department of Cardiology, Nanping First Hospital Affiliated to Fujian Medical University, Nanping, Fujian, China

**Keywords:** cytochrome P450, drug-drug interaction, heart failure, mavacamten, obstructive hypertrophic cardiomyopathy, pharmacovigilance

## Abstract

**Background:**

Mavacamten, a cardiac myosin inhibitor for symptomatic obstructive hypertrophic cardiomyopathy (HOCM), carries a risk of excessive negative inotropy. Because mavacamten is metabolized primarily by the cytochrome P450 (CYP) enzymes CYP2C19 and CYP3A4, concomitant CYP inhibitors may increase mavacamten exposure. We evaluated real-world heart failure-related reporting patterns associated with concomitant CYP inhibitor exposure in mavacamten-treated HOCM reports.

**Methods:**

We conducted a retrospective pharmacovigilance study using the U.S. Food and Drug Administration (FDA) Adverse Event Reporting System (FAERS) from the second quarter (Q2) of 2022 to the fourth quarter (Q4) of 2025. Mavacamten-treated HOCM reports were screened for concomitant exposure to ten prespecified CYP2C19 and/or CYP3A4 inhibitors. Heart failure-related signals were defined using seven Medical Dictionary for Regulatory Activities (MedDRA) Preferred Terms. Severe outcomes were extracted from FAERS outcome fields; hospitalization was defined as initial or prolonged hospitalization, and death as a reported fatal outcome. Analyses included the reporting odds ratio (ROR), additive signal amplification index (S), time-to-onset modeling, multivariable logistic regression, and 1:4 propensity score matching (PSM).

**Results:**

Among 3,570 mavacamten-treated HOCM reports, 347 involved concomitant CYP inhibitor exposure. Combination therapy showed an elevated heart failure-related reporting signal (ROR = 25.08; 95% confidence interval [CI], 18.63–33.76) and potential reporting signal amplification (S = 2.71). Agent-specific reporting signals were heterogeneous across prespecified inhibitors. Weibull modeling showed an early-failure pattern (β = 0.88; median onset, 80 days). Compared with mavacamten monotherapy, combination therapy had higher reported proportions of hospitalization (28.5% vs. 13.5%) and death (5.5% vs. 2.3%). Concomitant CYP inhibitor exposure showed higher heart failure-related reporting odds after multivariable adjustment (adjusted odds ratio [aOR], 2.52; 95% CI, 1.10–5.62) and in the PSM sensitivity cohort (aOR, 1.99; *P* < 0.001).

**Conclusion:**

Concomitant use of mavacamten with selected CYP inhibitors was associated with increased heart failure-related reporting in FAERS. Careful medication reconciliation and clinical vigilance are warranted, while recognizing that spontaneous reporting data cannot establish causality.

## 1 Introduction

Obstructive hypertrophic cardiomyopathy (HOCM) is a prevalent genetic cardiovascular disorder defined by left ventricular hypertrophy, hypercontractility, and dynamic left ventricular outflow tract (LVOT) obstruction (Ostrominski et al., 2023). Historically, the pharmacological management of HOCM has relied on non-specific agents, including beta-blockers and calcium channel blockers, which often provide incomplete symptom relief without directly targeting the underlying disease mechanism (Garcia-Pavia et al., 2025).

Mavacamten is a first-in-class, orally administered, selective allosteric inhibitor of cardiac myosin adenosine triphosphatase (ATPase) that directly addresses the molecular pathophysiology of HOCM (Quarta et al., 2026). By reducing excessive actin–myosin cross-bridge formation, mavacamten decreases myocardial contractility and alleviates LVOT obstruction (Nag et al., 2023). Landmark clinical trials, including EXPLORER-HCM (Clinical Study to Evaluate Mavacamten [MYK-461] in Adults With Symptomatic Obstructive Hypertrophic Cardiomyopathy) and VALOR-HCM (A Study to Evaluate Mavacamten in Adults With Symptomatic Obstructive Hypertrophic Cardiomyopathy Who Are Eligible for Septal Reduction Therapy), demonstrated significant improvements in exercise capacity, LVOT gradients, symptom burden, and cardiac remodeling among patients with symptomatic obstructive disease (He et al., 2021; Desai et al., 2024). A recent meta-analysis further confirmed its favorable effects on functional status and cardiac biomarkers (Aman et al., 2025). Despite these therapeutic benefits, the mechanism responsible for clinical efficacy—targeted negative inotropy—may also increase the risk of systolic dysfunction and heart failure-related adverse events (Desai et al., 2025). In the EXPLORER-LTE study, transient reductions in left ventricular ejection fraction (LVEF) below 50% and cardiac failure events were reported during long-term follow-up (Rader et al., 2024). Consequently, mavacamten therapy requires careful surveillance through structured echocardiographic monitoring and Risk Evaluation and Mitigation Strategy (REMS) programs (Desai et al., 2025).

An important vulnerability of mavacamten is its dependence on cytochrome P450 metabolism. Approximately 74% of mavacamten metabolism is mediated through CYP2C19 and about 18% through CYP3A4 (Chiang et al., 2023). In routine clinical practice, patients with HOCM frequently have concomitant cardiovascular and systemic comorbidities, including hypertension, atrial fibrillation, and gastroesophageal disorders, requiring additional pharmacotherapy (Wang et al., 2024; Philipson et al., 2021). Many commonly prescribed medications, including proton-pump inhibitors, calcium-channel blockers, antiarrhythmic agents, azole antifungals, and macrolide antibiotics, act as CYP2C19 and/or CYP3A4 inhibitors and may increase systemic mavacamten exposure, thereby potentially enhancing its negative inotropic effects and contributing to reports of systolic dysfunction and heart failure-related outcomes (Chiang et al., 2023; Reyes et al., 2022).

Although prescribing information highlights the potential for clinically important CYP-mediated drug–drug interactions (DDIs), the real-world consequences of concomitant CYP inhibitor use in mavacamten-treated patients—including the extent of heart failure-related reporting, timing of reported onset, and patterns of reported clinical severity—have not been systematically characterized in large pharmacovigilance datasets (Reza et al., 2025; Yukselen et al., 2024; DeVries et al., 2023).

To address this knowledge gap, we conducted a comprehensive pharmacovigilance study using the U.S. Food and Drug Administration (FDA) Adverse Event Reporting System (FAERS) database. By integrating established pharmacovigilance methods including disproportionality analyses for signal detection, time-to-onset modeling, multivariable logistic regression, and propensity score matching (PSM), we systematically evaluated the clinical severity, signal amplification, and covariate-adjusted association of concomitant CYP inhibitor exposure in mavacamten-treated patients with HOCM. Similar real-world FAERS pharmacovigilance approaches have been successfully applied to characterize the safety profile of mavacamten in recent observational studies (Yukselen et al., 2024).

## 2 Methods

### 2.1 Study design and data source

A retrospective pharmacovigilance study was conducted using data extracted from the FAERS database (F.A.E.R.S. FAERS, 2023). To capture the post-marketing safety profile of mavacamten following its approval, data spanning from the second quarter (Q2) of 2022 to the fourth quarter (Q4) of 2025 were retrieved. To ensure data integrity and mitigate reporting bias, a rigorous data cleaning and deduplication process was performed prior to analysis, strictly adhering to the FDA’s recommendations. Specifically, for reports sharing the same CASEID, the report with the most recent FDA_DT (FDA receive date) and the highest PRIMARYID was retained. In FAERS, CASEID represents a case identifier, PRIMARYID represents a unique report identifier for a specific version of a case report, and FDA_DT represents the FDA receipt date.

### 2.2 Cohort identification and exposure definition

The primary study population was strictly restricted to FAERS reports with a documented indication of HOCM. Within this disease-specific cohort, patients with recorded exposure to mavacamten were identified. To evaluate reporting patterns associated with concomitant CYP inhibitor exposure, the mavacamten-exposed cohort was stratified into two non-overlapping treatment subgroups.

1. Mavacamten Monotherapy Group: Patients receiving Mavacamten without the concurrent use of any targeted CYP inhibitors.
2. Combination Therapy Group: Patients receiving Mavacamten concomitantly with a prespecified list of 10 established CYP inhibitors targeting the CYP2C19 and CYP3A4 metabolic pathways, including omeprazole, esomeprazole, fluconazole, fluvoxamine, itraconazole, ketoconazole, clarithromycin, diltiazem, verapamil, and amiodarone. These ten inhibitors were prespecified as a hypothesis-driven, clinically oriented set based on their potential relevance to mavacamten-treated patients, established inhibitory effects on CYP2C19 and/or CYP3A4, and available mavacamten-specific or class-level pharmacokinetic and drug–drug interaction evidence (Chiang et al., 2023; Perera et al., 2023; Mar et al., 2022; Ogilvie et al., 2011). The selected agents were intended to represent clinically relevant exposures rather than an exhaustive list of all known CYP2C19 or CYP3A4 inhibitors.

### 2.3 Adverse event ascertainment and severity classification

The primary outcome was a composite of heart failure-related reports identified using seven prespecified Medical Dictionary for Regulatory Activities (MedDRA) Preferred Terms (PTs): cardiac failure, cardiac failure congestive, cardiac failure acute, ejection fraction decreased, systolic dysfunction, left ventricular dysfunction, and acute pulmonary oedema (Supplementary Table S1) (Brown et al., 1999). The selected PTs were designed to capture clinically reported heart failure, systolic dysfunction, reduced ejection fraction, and pulmonary congestion events potentially related to excessive negative inotropy during mavacamten therapy. Reported clinical severity was assessed using FAERS outcome information, including death, life-threatening events, and hospitalization (initial or prolonged). These outcomes were analyzed as severity indicators and were not components of the primary heart failure-related composite endpoint.

### 2.4 Disproportionality analysis and exploratory signal amplification assessment

In this study, adverse event (AE), adverse drug event (ADE), and adverse drug reaction (ADR) refer to reported events in FAERS rather than adjudicated causal reactions. To quantitatively detect and validate the heart failure-related reporting signal, a comprehensive disproportionality analysis was executed based on a 2 × 2 contingency table. The parameters in the table are defined as follows: ‘a’ represents the number of reports containing both the target drug and the target ADR; ‘b’ represents the number of reports containing the target drug but other adverse drug reactions; ‘c’ represents the number of reports containing the target adverse drug reaction but other drugs; ‘d’ represents the number of reports containing neither the target drug nor the target adverse drug reaction.

For disproportionality analyses, a background comparator cohort was constructed using all FAERS reports from the same study period (Q2 2022 to Q4 2025) involving any of the ten prespecified CYP inhibitors in the absence of mavacamten exposure. This comparator cohort was used exclusively for signal detection and was not included in the primary HOCM cohort analyses.

To ensure signal robustness and align with established pharmacovigilance practice, three disproportionality algorithms—encompassing both frequentist and Bayesian methodologies—were employed.

1. Reporting Odds Ratio (ROR) (Rothman et al., 2004): The ROR, primarily utilized by the Netherlands Pharmacovigilance Centre Lareb, compares the odds of reporting a specific ADE for a specific drug with the odds for all other drugs. A signal is generated when *a* ≥ 3 and the lower bound of the 95% confidence interval (CI) is ≥1. The computational formulas are:

$$ROR=\frac{a/b}{c/d}$$

$$SE\left(\ln ROR\right)=\sqrt{\frac{1}{a}+\frac{1}{b}+\frac{1}{c}+\frac{1}{d}}$$

$$95\%\;CI=e^{\ln\left(ROR\right)\pm 1.96\;\sqrt{\left(\frac{1}{a}+\frac{1}{b}+\frac{1}{c}+\frac{1}{d}\right)}}$$

2. Proportional Reporting Ratio (PRR) (Noguchi et al., 2020): Originally developed and adopted by the United Kingdom Medicines and Healthcare products Regulatory Agency (MHRA), the PRR compares the proportion of reports containing a specific ADE for the target drug with the corresponding reporting proportion for all other drugs in the database. A signal is generated when *a* ≥ 3 and PRR ≥2. The formulas are:

$$PRR=\frac{a/\left(a+b\right)}{c/\left(c+d\right)}$$

$$SE\left(\ln PRR\right)=\sqrt{\left(\frac{1}{a}-\frac{1}{a+b}+\frac{1}{c}-\frac{1}{c+d}\right)}$$

$$95\%CI=e^{\ln\left(PRR\right)\pm 1.96\sqrt{\left(\frac{1}{a}-\frac{1}{a+b}+\frac{1}{c}-\frac{1}{c+d}\right)}}$$

3. Bayesian Confidence Propagation Neural Network (BCPNN) (Tada et al., 2020): Implemented by the World Health Organization Uppsala Monitoring Centre (WHO-UMC), the BCPNN serves as an important tool for handling uncertain information by calculating the Information Component (IC). A signal is established when the lower limit of the 95% credible interval (IC-2SD, or IC025) is >0. The formulas are:

$$IC=\log_{2}\frac{a\left(a+b+c+d\right)}{\left(a+c\right)\left(a+b\right)}$$

$$E\left(IC\right)=\log_{2}\frac{\left(a+\gamma 11\right)\left(a+b+c+d+\alpha \right)\left(a+b+c+d+\beta \right)}{\left(a+b+c+d+\gamma \right)\left(a+b+\alpha 1\right)\left(a+c+\beta 1\right)}$$

$$V\left(IC\right)=\frac{1}{{\left(\ln2\right)}^{2}}\left\{\left[\frac{\left(a+b+c+d\right)-a+\gamma +\gamma 11}{\left(a+\gamma 11\right)\left(1+a+b+c+d+\gamma \right)}\right]+\left[\frac{\left(a+b+c+d\right)-\left(a+b\right)+\alpha -\alpha 1}{\left(a+b+\alpha 1\right)\left(1+a+b+c+d+\alpha \right)}\right]+\left[\frac{\left(a+b+c+d\right)-\left(a+c\right)+\beta -\beta 1}{\left(a+c+\beta 1\right)\left(1+a+b+c+d+\beta \right)}\right]\right\}$$

$$\gamma =\gamma 11\frac{\left(a+b+c+d+\alpha \right)\left(a+b+c+d+\beta \right)}{\left(a+b+\alpha 1\right)\left(a+c+\beta 1\right)}$$

$$IC-2SD=E\left(IC\right)-2V{\left(IC\right)}^{0.5}$$

Furthermore, to explore whether the reporting signal observed in the combination group exceeded a simple additive reporting pattern, an exploratory additive signal amplification index (S) was calculated based on ROR values, using an odds-ratio-based additive interaction framework as a reference (Kalilani and Atashili, 2006). Because ROR is a disproportionality measure rather than a biological risk estimate, this analysis was interpreted only as reporting signal amplification rather than pharmacological interaction, biological interaction, or clinical risk.

### 2.5 Time-to-onset (TTO) and hazard dynamics modeling

The TTO of heart failure-related reports was calculated as the interval (in days) between the initiation of the therapeutic regimen and the reported onset date. To delineate the precise temporal hazard pattern, a Weibull distribution model was fitted to the TTO data. The Weibull shape parameter (β) was utilized to characterize the failure rate over time.

1. β < 1 indicates an “early failure” hazard type (risk peaks sharply upon initial exposure and subsequently decreases).
2. β = 1 indicates a random failure hazard type (constant risk).
3. β > 1 indicates a wear-out failure hazard type (risk increases over time due to chronic accumulation).

### 2.6 Statistical analysis

Descriptive statistics were utilized to summarize baseline demographic and clinical characteristics. Continuous variables were compared using the Mann-Whitney U test, while categorical variables were analyzed using Pearson’s chi-square test or Fisher’s exact test, as appropriate. To evaluate factors associated with heart failure-related reporting and account for measured baseline clinical imbalances, a multivariable logistic regression model was constructed. All statistical analyses were two-sided, and a *P* < 0.05 was considered statistically significant. Data management and statistical computing were performed using R software (version 4.4.1).

In response to the concern that dichotomizing age and body weight may introduce residual confounding, we additionally performed a complete-case sensitivity analysis in which age and body weight were entered as continuous variables. This model adjusted for concomitant CYP inhibitor use, continuous age, continuous body weight, and sex. Because body weight was missing in a substantial proportion of FAERS reports, this analysis was considered sensitivity analysis only and was not used to replace the primary model.

Given the inherently hypothesis-generating nature of pharmacovigilance data mining, all subgroup and agent-specific analyses were predefined as exploratory. Consequently, no formal multiplicity adjustments (e.g., Bonferroni correction) were applied to the confidence intervals or *P*-values. These secondary findings should be interpreted as signal-generating rather than confirmatory, and warrant further prospective validation.

## 3 Results

### 3.1 Study population and baseline characteristics

**FIGURE 1:**
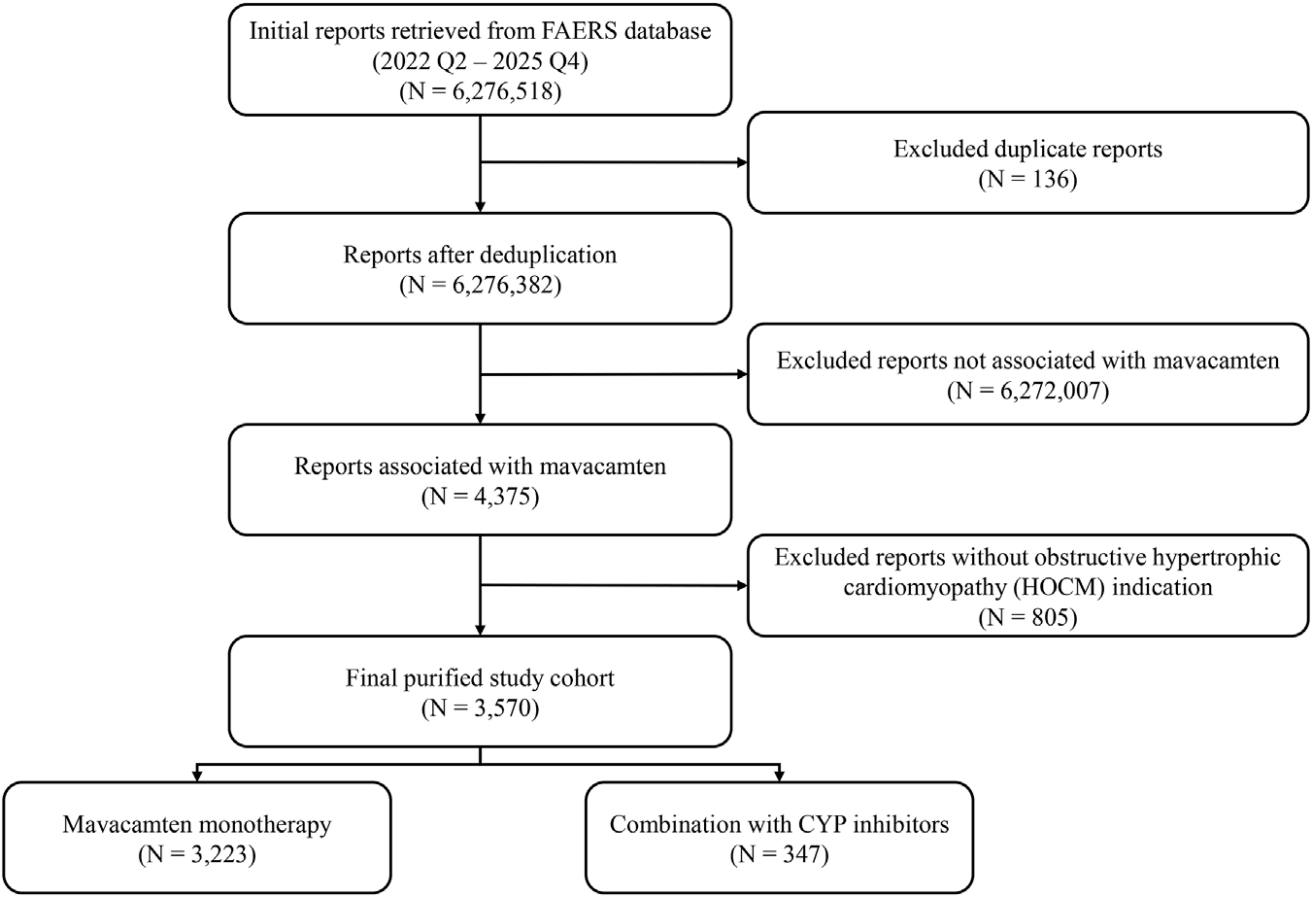
Study selection flowchart. Flowchart detailing the data extraction, deduplication, and cohort identification process from the FAERS database (Q2 2022 to Q4 2025). A final cohort of 3,570 mavacamten-treated HOCM reports was identified and subsequently stratified into two sub-cohorts: the mavacamten monotherapy group (n = 3,223) and the concomitant CYP inhibitor therapy group (n = 347). Abbreviations: FAERS, FDA Adverse Event Reporting System; HOCM, obstructive hypertrophic cardiomyopathy; CYP, cytochrome P450.

As detailed in the study selection flowchart (Figure 1), after rigorous deduplication and restriction to patients with HOCM, a final cohort of 3,570 mavacamten-treated HOCM reports was identified from the FAERS database. These reports were stratified into a mavacamten monotherapy group (N = 3,223) and a concomitant CYP inhibitor therapy group (N = 347).

Baseline demographic and clinical characteristics of the overall study cohort are summarized in Table 1. Demographically, the combination therapy group had a higher proportion of elderly patients (≥65 years: 57.1% vs. 50.0%) and males (30.8% vs. 26.2%) compared to the monotherapy group (all *P* < 0.001). Furthermore, a significantly larger fraction of reports in the combination group was submitted by healthcare professionals (35.7% vs. 21.9%, *P* < 0.001).

**TABLE 1:** Baseline characteristics and clinical outcomes of the study cohort.

Characteristics	Mavacamten monotherapy (n = 3223)	Combination with CYP inhibitors (n = 347)	*P* value
Gender, n (%)	​	​	<0.001
Female	1963 (60.9%)	222 (64.0%)	​
Male	844 (26.2%)	107 (30.8%)	​
Unknown	416 (12.9%)	18 (5.2%)	​
Age, n (%), years	​	​	<0.001
<65 years	924 (28.7%)	129 (37.2%)	​
≥65	1612 (50.0%)	198 (57.1%)	​
Unknown	687 (21.3%)	20 (5.8%)	​
Weight, n (%), kg	​	​	<0.001
<60	38 (1.2%)	20 (5.8%)	​
≥60	298 (9.2%)	70 (20.2%)	​
Unknown	2887 (89.6%)	257 (74.1%)	​
Reporter, n (%)	​	​	<0.001
HCP	705 (21.9%)	124 (35.7%)	​
Non-HCP	1691 (52.5%)	118 (34.0%)	​
Unknown	827 (25.7%)	105 (30.3%)	​
Atrial fibrillation, n (%)	26 (0.8%)	25 (7.2%)	<0.001
Diabetes mellitus, n (%)	13 (0.4%)	4 (1.2%)	0.072
Beta-blockers, n (%)	618 (19.2%)	189 (54.5%)	<0.001
Diuretics, n (%)	210 (6.5%)	110 (31.7%)	<0.001
RAS inhibitors/ARNI, n (%)	193 (6.0%)	88 (25.4%)	<0.001
Clinical outcomes, n (%)			
Hospitalization	435 (13.5%)	99 (28.5%)	<0.001
Death	75 (2.3%)	19 (5.5%)	0.002
Life-threatening	49 (1.5%)	17 (4.9%)	<0.001

Data are presented as n (%). Differences between groups were evaluated using Pearson’s chi-square test or Fisher’s exact test, as appropriate. Abbreviations: CYP, cytochrome P450; HCP, healthcare professional; RAS, renin-angiotensin system; ARNI, angiotensin receptor-neprilysin inhibitor.

Regarding clinical comorbidities and concurrent medications, patients receiving combination therapy exhibited a substantially higher baseline cardiovascular disease burden. Specifically, the prevalence of atrial fibrillation was remarkably elevated in the combination cohort (7.2% vs. 0.8%, *P* < 0.001), whereas the difference in diabetes mellitus did not reach statistical significance (1.2% vs. 0.4%, *P* = 0.072).

Additionally, the combination group had significantly higher concomitant use of cardiovascular medications, including beta-blockers, diuretics, and renin–angiotensin system inhibitors or angiotensin receptor–neprilysin inhibitors (all *P* < 0.001).

Notably, an initial analysis of severe clinical outcomes within the overall cohort showed higher reported clinical severity in the combination group. Reports involving concomitant CYP inhibitor exposure had higher reported proportions of hospitalization (28.5% vs. 13.5%, *P* < 0.001), death (5.5% vs. 2.3%, *P* = 0.002), and life-threatening outcomes (4.9% vs. 1.5%, *P* < 0.001) than reports involving mavacamten alone.

### 3.2 Disproportionality analysis and exploratory signal amplification assessment

To ensure the robustness of signal detection, disproportionality analyses were conducted using three complementary algorithms encompassing both frequentist and Bayesian methodologies (Table 2). The results demonstrated a substantial increase in the reporting signal for heart failure-related reports associated with concomitant CYP inhibitor exposure. Mavacamten monotherapy demonstrated a moderate positive signal for heart failure-related reports (ROR = 7.77, 95% CI: 6.64–9.10; information component [IC] = 2.89, with the lower limit of the 95% credible interval [IC025] > 0), whereas the background comparator cohort consisting of reports involving CYP inhibitors without concomitant mavacamten exposure exhibited only a weak baseline signal (ROR = 3.12, 95% CI: 3.02–3.22; IC = 1.52, IC025 > 0). In contrast, the combination therapy group demonstrated markedly stronger disproportionality signals across all signal-detection metrics, with an ROR of 25.08 (95% CI: 18.63–33.76), a PRR of 21.54, and an IC of 4.43 (IC025 > 0).

**TABLE 2:** Disproportionality analysis of heart failure-related reporting signals across different treatment regimens.

Therapeutic regimen	Total reports (N)	Heart failure-related reports (N)	ROR (95% CI)	PRR	IC (95% CrI)	Signal amplification index (S)
Mavacamten monotherapy	3223	163	7.77 (6.64–9.10)	7.43	2.89 (2.67–3.11)	—
CYP inhibitors without mavacamten	212783	4175	3.12 (3.02–3.22)	3.07	1.52 (1.48–1.57)	—
Combination (mavacamten + inhibitor)	347	51	25.08 (18.63–33.76)	21.54	4.43 (4.03–4.82)	2.71

The CYP inhibitor comparator cohort included FAERS reports involving any of the ten prespecified CYP inhibitors without concomitant mavacamten exposure and was used solely for disproportionality signal estimation. This cohort was not part of the primary HOCM study population. A positive safety signal was defined according to the following established thresholds: lower limit of the 95% CI, for ROR ≥1; PRR ≥2; and IC025 > 0. Abbreviations: CYP, cytochrome P450; ROR, reporting odds ratio; CI, confidence interval; PRR, proportional reporting ratio; IC, information component; CrI, credible interval; IC025, lower limit of the 95% credible interval for the IC.

To further evaluate whether the observed reporting signal amplification exceeded a simple additive reporting pattern, an additive signal amplification index (S) was calculated. In the present study, a supra-additive reporting signal refers to a reporting signal that exceeds the expected additive reporting pattern of mavacamten exposure and CYP inhibitor exposure alone, as quantified by this exploratory index. Given the relatively limited signal associated with CYP inhibitor reports without mavacamten, the resulting index of S = 2.71 (S > 1) supported the presence of potential supra-additive reporting signal amplification. This finding should be interpreted as reporting signal amplification rather than evidence of a biological interaction, pharmacological interaction, or increased clinical risk.

**FIGURE 2:**
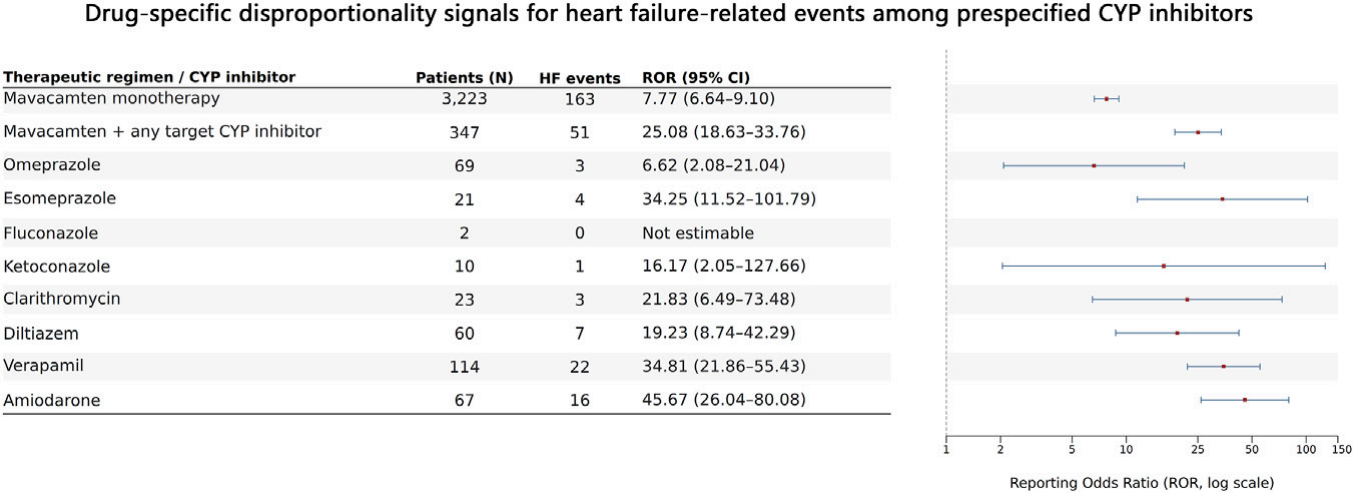
Drug-specific disproportionality signals for heart failure-related reports among prespecified CYP inhibitors. Forest plot showing reporting odds ratios (RORs) and 95% confidence intervals (CIs). Drug-specific counts correspond to Supplementary Table S2. Rows are not mutually exclusive because individual reports may contain more than one CYP inhibitor. Abbreviations: CYP, cytochrome P450; ROR, reporting odds ratio; CI, confidence interval.

Furthermore, drug-specific disproportionality analysis based on the locked report-level CYP inhibitor distribution identified heterogeneity across individual agents (Figure 2). Among the prespecified CYP inhibitors, the strongest reporting signals were observed for amiodarone (67 reports; 16 heart failure-related reports; ROR = 45.67, 95% CI: 26.04–80.08), verapamil (114 reports; 22 heart failure-related reports; ROR = 34.81, 95% CI: 21.86–55.43), esomeprazole (21 reports; 4 heart failure-related reports; ROR = 34.25, 95% CI: 11.52–101.79), clarithromycin (23 reports; 3 heart failure-related reports; ROR = 21.83, 95% CI: 6.49–73.48), diltiazem (60 reports; 7 heart failure-related reports; ROR = 19.23, 95% CI: 8.74–42.29), ketoconazole (10 reports; 1 heart failure-related report; ROR = 16.17, 95% CI: 2.05–127.66), and omeprazole (69 reports; 3 heart failure-related reports; ROR = 6.62, 95% CI: 2.08–21.04). Fluconazole had no heart failure-related reports and was therefore not estimable. The complete distribution of prespecified CYP inhibitors and corresponding heart failure-related report counts is presented in Supplementary Table S2.

Among the combination therapy reports, 31 involved simultaneous exposure to more than one CYP inhibitor. Heart failure-related reports occurred in 5 of these reports (16.1%), compared with 42 heart failure-related reports among 290 reports involving a single CYP inhibitor (14.5%). Detailed results are presented in Supplementary Table S3.

### 3.3 Temporal dynamics and reported clinical severity

**FIGURE 3:**
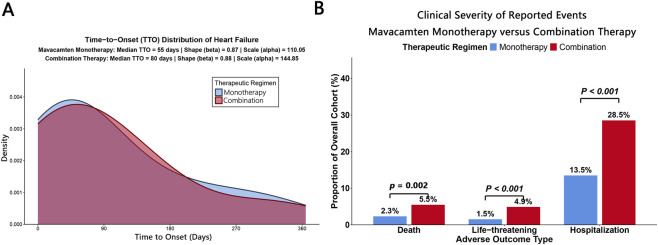
Temporal dynamics and reported clinical severity of heart failure-related reports. **(A)** TTO distribution fitted with a Weibull hazard model. Both the mavacamten monotherapy (β = 0.87) and combination therapy (β = 0.88) groups exhibited an “early failure” hazard profile (β < 1). **(B)** Comparison of severe reported outcomes between reports involving mavacamten monotherapy and reports involving concomitant CYP inhibitor exposure. Abbreviations: TTO, time-to-onset; CYP, cytochrome P450; β, Weibull shape parameter.

To characterize the temporal dynamics and reported clinical severity of heart failure-related reports, Figure 3 illustrates both the TTO distribution and severe reported outcomes.

As depicted in Figure 3A, a Weibull distribution model was applied to delineate the temporal pattern of reported onset. In the mavacamten monotherapy cohort, the median TTO was 55 days, which was consistent with the early treatment and dose-titration period during which negative inotropic intolerance may become clinically apparent. The combination therapy group demonstrated a median TTO of 80 days. The Weibull shape parameter (β) was less than 1 for both the monotherapy (β = 0.87) and combination (β = 0.88) groups. A β value below 1 is consistent with an “early failure” hazard pattern, suggesting that heart failure-related reporting was concentrated earlier after exposure and then decreased over time.

Furthermore, Figure 3B summarizes the distribution of severe reported outcomes by treatment group. Consistent with the overall cohort summary, reports involving concomitant CYP inhibitor exposure showed higher reported proportions of hospitalization, life-threatening outcomes, and death than reports involving mavacamten alone.

### 3.4 Multivariable logistic regression and reporting source assessment

**FIGURE 4:**
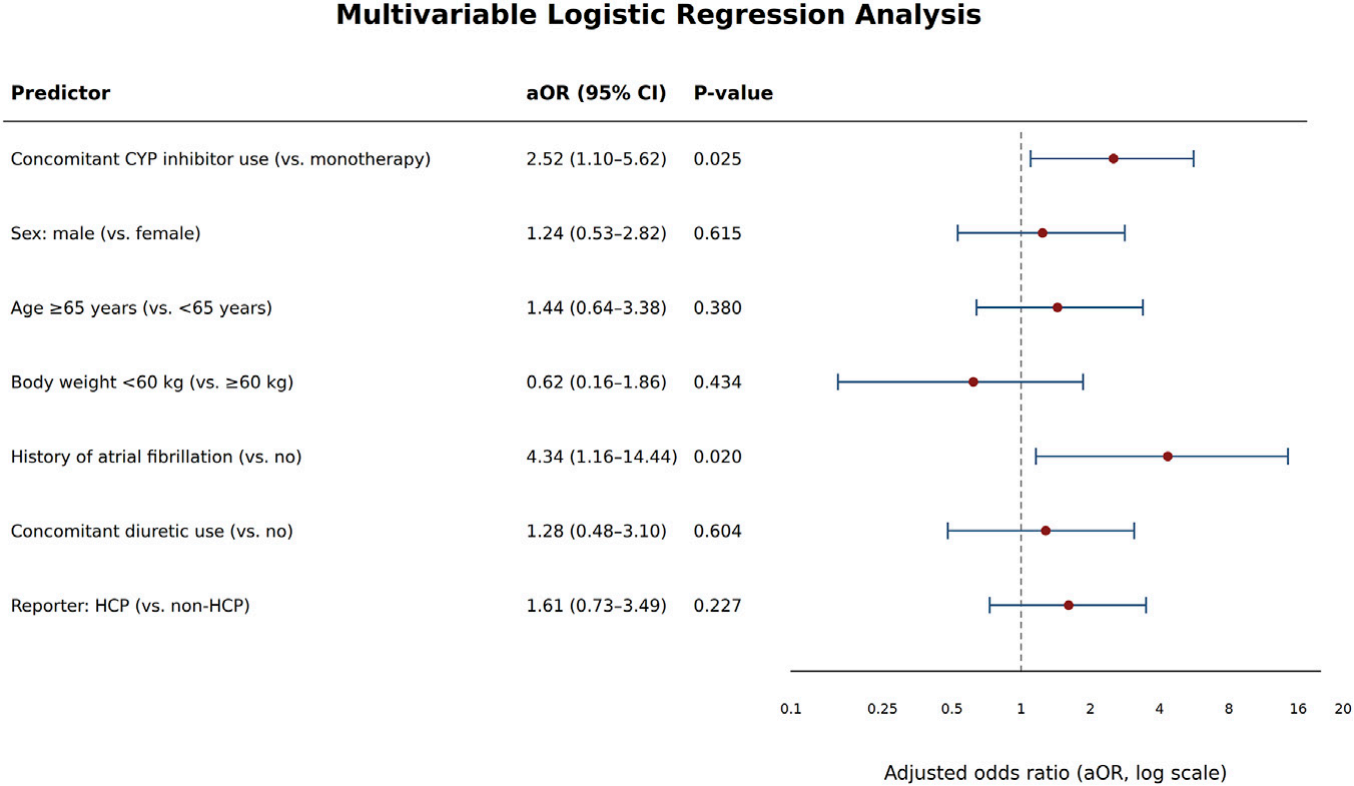
Multivariable logistic regression analysis of factors associated with heart failure-related reporting. The forest plot displays the aORs and 95% CIs on a logarithmic scale. The regression model adjusted for baseline clinical imbalances and potential reporting-related factors, including age (≥65 years), sex, low body weight (<60 kg), history of atrial fibrillation, concomitant diuretic use, and reporting source. Abbreviations: aOR, adjusted odds ratio; CI, confidence interval; HCP, healthcare professional.

To evaluate whether concomitant CYP inhibitor use was associated with heart failure-related reporting after accounting for baseline clinical imbalances and reporting source, a multivariable logistic regression analysis was conducted (Figure 4). The model incorporated and adjusted for critical demographic and clinical covariates, including age (≥65 years), sex, low body weight (<60 kg), history of atrial fibrillation, concomitant diuretic use, and specifically, the reporting source (healthcare professional [HCP] vs. non-HCP).

To address potential residual confounding introduced by categorizing age and body weight, we performed an additional complete-case sensitivity analysis in which age and body weight were modeled as continuous variables. In this analysis, concomitant CYP inhibitor use showed higher reporting odds for heart failure-related reports (OR = 3.13, 95% CI: 1.48–6.62, *P* = 0.00285), whereas continuous age (OR = 1.02, 95% CI: 0.99–1.06, *P* = 0.160) and body weight (OR = 1.00, 95% CI: 0.99–1.01, *P* = 0.837) were not significantly associated with the outcome. These findings support the robustness of the primary model and suggest that the observed association was not materially influenced by the categorization of age or body weight (Supplementary Table S4).

Following adjustment, concomitant CYP inhibitor exposure remained associated with increased heart failure-related reporting among mavacamten-treated HOCM reports. Specifically, combination therapy showed higher reporting odds compared with mavacamten monotherapy (adjusted odds ratio, aOR = 2.52; 95% CI: 1.10–5.62; *P* = 0.025). A history of atrial fibrillation was also associated with heart failure-related reporting in this cohort (aOR = 4.34; 95% CI: 1.16–14.44; *P* = 0.020). The reporting source (HCP) did not reach statistical significance as a predictor of heart failure-related reporting (*P* = 0.227), nor did other measured baseline covariates, including advanced age, male sex, low body weight, and concomitant diuretic use (all *P* > 0.05). These multivariable findings should be interpreted as reporting associations after adjustment for measured baseline covariates and reporting source, rather than evidence of a causal increase in clinical risk.

### 3.5 Sensitivity analysis via propensity score matching

To further evaluate the robustness of the primary multivariable logistic regression model and to account for baseline imbalances—particularly the different prevalences of atrial fibrillation and concomitant diuretic use—a 1:4 PSM analysis was performed as a sensitivity analysis. To preserve the integrity of the full study cohort (n = 3,570), missing baseline demographics were categorized as an independent “Unknown” tier during the matching process.

**FIGURE 5:**
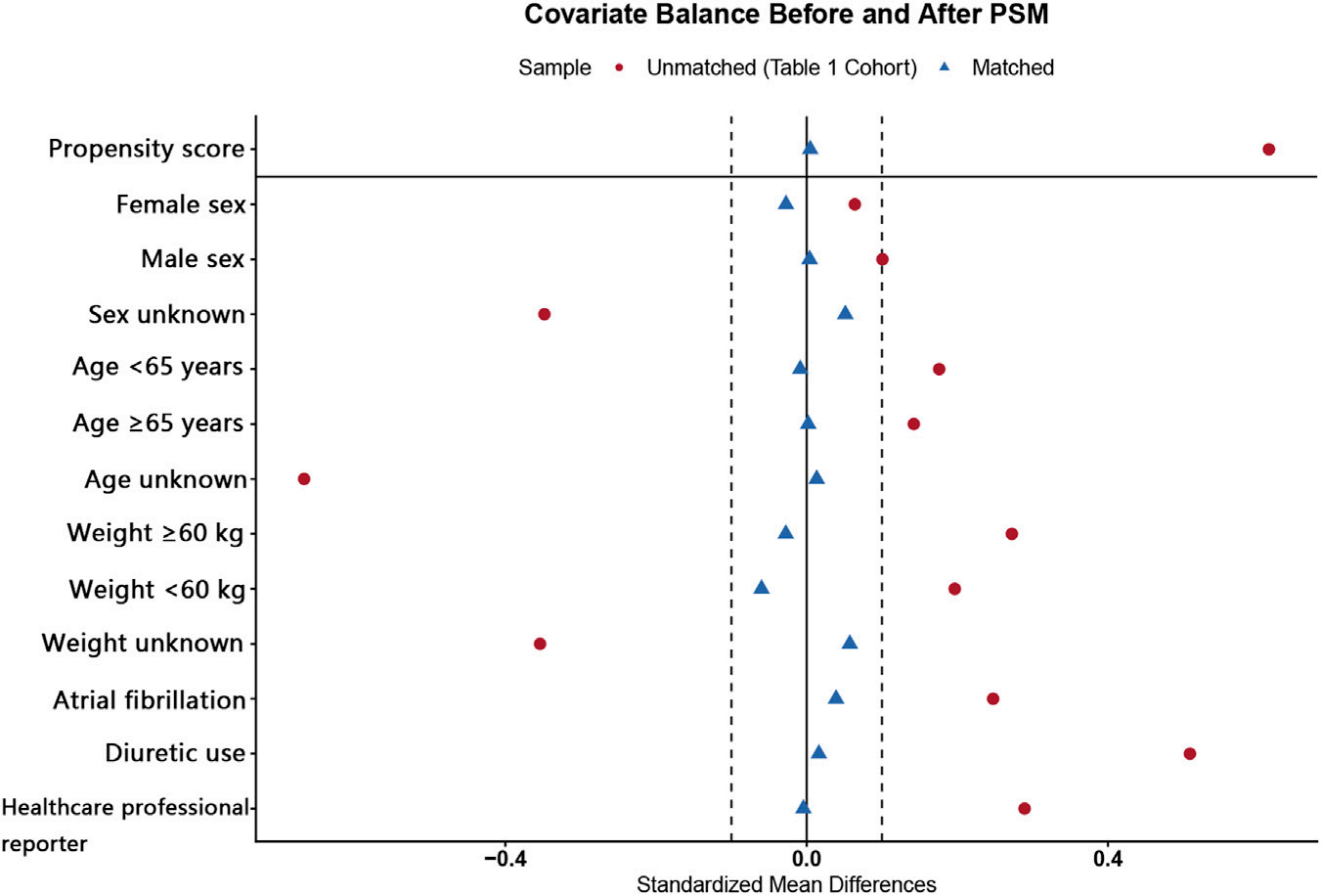
Covariate balance before and after PSM. The Love plot displays the SMDs of baseline covariates between the mavacamten monotherapy group and the concomitant CYP inhibitor therapy group. Red circles indicate the unmatched cohort, whereas blue triangles represent the 1:4 propensity score–matched cohort. Vertical dashed lines denote the prespecified balance threshold of SMD = ±0.1. Abbreviations: PSM, propensity score matching; SMD, standardized mean difference; CYP, cytochrome P450.

The PSM algorithm successfully achieved adequate covariate balance across all included baseline and reporting covariates, with the standardized mean differences (SMDs) for all matched variables—including the independently categorized missing data tiers—constrained well below the rigorous threshold of 0.1 (Figure 5). Consistent with the primary multivariable findings, analysis of the matched cohort showed higher heart failure-related reporting odds in reports involving concomitant CYP inhibitor exposure (aOR = 1.99; 95% CI: 1.37–2.88; *P* < 0.001; Supplementary Table S5). These findings provided a sensitivity analysis showing a consistent association after covariate balancing.

## 4 Discussion

To our knowledge, this is the first real-world pharmacovigilance study to evaluate heart failure-related reporting associated with concomitant mavacamten and selected CYP inhibitor use. The combination cohort showed a stronger reporting signal than mavacamten monotherapy, and the association remained consistent after multivariable adjustment and propensity score matching. Nevertheless, these findings are based on spontaneous reports and should be interpreted as signal-generating rather than causal estimates of clinical risk.

The observed association is mechanistically plausible because mavacamten is primarily metabolized by CYP2C19 and CYP3A4 (Chiang et al., 2023). CYP2C19 and/or CYP3A4 inhibition may increase mavacamten exposure and enhance its negative inotropic effects (Mar et al., 2022; Ricci et al., 2025; Merali et al., 2025). This study intentionally used a targeted, hypothesis-driven list of ten inhibitors selected on the basis of their potential clinical relevance to mavacamten-treated patients, established inhibition of CYP2C19 and/or CYP3A4, and available mavacamten-specific or class-level pharmacokinetic and drug–drug interaction evidence. An untargeted screen of all possible CYP2C19 and CYP3A4 inhibitors was not undertaken because inclusion of numerous additional agents would likely generate sparse drug-specific strata and unstable estimates in spontaneous reporting data. Although agent-specific reporting signals varied across the prespecified inhibitors, overlapping inhibitor exposure and small drug-specific strata precluded reliable comparisons or ranking of individual agents. Accordingly, the agent-specific findings should be considered exploratory and hypothesis-generating, and their generalizability to other CYP2C19 or CYP3A4 inhibitors is limited. The additive signal amplification index should also be interpreted cautiously. Because it was calculated from RORs derived from spontaneous reporting data, it reflects reporting signal amplification rather than biological interaction, pharmacological interaction, or a quantitative estimate of clinical risk. Other clinically important CYP2C19 and/or CYP3A4 inhibitors, such as ritonavir, cobicistat, erythromycin, posaconazole, and voriconazole, were not included because they were outside the prespecified clinically oriented exposure set. Expanding the analysis to additional inhibitors would likely generate sparse drug-specific strata and unstable estimates in the current FAERS-based dataset. Their exclusion should not be interpreted as evidence of absence of interaction potential, and future studies should evaluate these and other CYP-modulating agents in larger pharmacovigilance datasets and longitudinal clinical cohorts.

Confounding by indication remains a major limitation of this study. Reports in the combination cohort showed a greater baseline cardiovascular comorbidity and co-medication burden, including more frequent atrial fibrillation, beta-blocker use, diuretic use, renin–angiotensin system inhibitor or angiotensin receptor–neprilysin inhibitor use, hospitalization, and healthcare professional reporting. Although multivariable adjustment and propensity score matching reduced measured imbalance, important clinical confounders were unavailable in FAERS, including New York Heart Association (NYHA) functional class, LVOT gradient, baseline and serial LVEF, mavacamten dose, dose adjustment, renal function, liver disease, CYP2C19 genotype, duration of therapy, and medication adherence. Therefore, residual confounding may explain part of the observed reporting association, and the findings should not be interpreted as evidence of a causal increase in clinical heart failure risk.

These findings have practical implications for the clinical management of HOCM. The current FDA REMS program mandates routine echocardiographic surveillance for LVEF reductions (Desai et al., 2025). These reporting associations support careful medication reconciliation before and during mavacamten therapy, in addition to protocol-guided echocardiographic monitoring.

The present FAERS analysis could not evaluate the utility of serial BNP or NT-proBNP monitoring because biomarker data were not systematically available (Potter et al., 2025). Although natriuretic peptide measurements may provide complementary clinical information when indicated, evidence supporting their routine use for safety monitoring during mavacamten therapy remains limited. Therefore, protocol-guided echocardiographic assessment of LVEF remains the established monitoring approach (Desai et al., 2025). Concomitant use of amiodarone and mavacamten may result in a clinically significant drug–drug interaction and should be undertaken with caution, with close monitoring for reduced systolic function and other adverse cardiac effects (Ricci et al., 2025). For routine gastroprotection, clinicians should carefully review proton pump inhibitor selection and consider the CYP2C19 inhibitory potential of individual agents. Alternative proton pump inhibitors with lower CYP2C19 inhibitory potential, such as pantoprazole, may be considered when clinically appropriate (Ogilvie et al., 2011).

Despite the rigorous methodological design, this study has several limitations inherent to FAERS. First, FAERS is a spontaneous reporting system subject to underreporting, reporting bias, and absence of a true exposed population denominator; therefore, the proportions reported here represent reporting frequencies rather than absolute incidence rates (Cutroneo et al., 2023). Second, important clinical variables were unavailable or incompletely captured in FAERS, including NYHA functional class, LVOT gradient, baseline and serial LVEF, mavacamten dose and dose adjustment, renal function, liver disease, CYP2C19 pharmacogenetic phenotype, CYP inhibitor strength, medication timing and duration, mavacamten plasma concentrations, medication adherence, and clinical triggers for heart failure-related reporting such as supraventricular arrhythmia burden or volume status (Potter et al., 2025). The absence of exposure measurements precluded direct confirmation of drug accumulation or overdose. Although multivariable regression and PSM were used to reduce measured confounding, residual confounding from unmeasured disease severity, prescribing patterns, and monitoring intensity cannot be excluded. Third, FAERS does not reliably distinguish chronic continuous CYP inhibitor use from intermittent or on-demand use, such as chronic *versus* occasional omeprazole exposure. Fourth, the analysis was restricted to ten prespecified inhibitors; therefore, the findings should not be generalized to all CYP2C19/CYP3A4 inhibitors. Important inhibitors such as ritonavir, cobicistat, erythromycin, posaconazole, and voriconazole were not evaluated in the present analysis. CYP2C19/CYP3A4 inducers were not evaluated because the prespecified objective of this study focused on heart failure-related reporting potentially associated with increased mavacamten exposure during CYP inhibition. Published physiologically based pharmacokinetic modeling indicates that moderate-to-strong CYP2C19 or CYP3A4 inducers may reduce mavacamten exposure (Chiang et al., 2023). Future pharmacovigilance studies should evaluate both loss-of-effect signals during inducer coadministration and outcomes associated with changes in inducer therapy. Finally, heart failure-related reporting frequencies in FAERS should not be directly compared with event rates from pivotal trials. Real-world reports may involve patients with greater comorbidity burden, polypharmacy, variable adherence, and less standardized monitoring, whereas pivotal trials used strict eligibility criteria, protocol-guided LVEF surveillance, dose adjustment, and structured adverse event ascertainment. These differences may partly explain the higher apparent frequency of heart failure-related reports in this pharmacovigilance dataset. Future studies using longitudinal clinical datasets are needed to validate the agent-specific signals and evaluate a broader range of CYP-modulating drugs.

## 5 Conclusion

In conclusion, this pharmacovigilance study identified increased heart failure-related reporting associated with concomitant use of mavacamten and selected CYP inhibitors in HOCM reports. Consistent findings across disproportionality analysis, time-to-onset modeling, multivariable regression, and propensity score-matched analysis support the robustness of this reporting signal, although causality and true clinical risk cannot be established from spontaneous reporting data. Clinical interpretation should therefore emphasize careful medication reconciliation, review of CYP-interacting drugs before treatment initiation and during dose titration, and protocol-guided echocardiographic surveillance of LVEF. BNP or NT-proBNP measurement may be considered as an adjunct when clinically indicated, but it should not replace echocardiographic assessment.

## Data Availability

Publicly available datasets were analyzed in this study. This data can be found here: U.S. Food and Drug Administration Adverse Event Reporting System (FAERS) Quarterly Data Extract Files: https://fis.fda.gov/extensions/FPD-QDE-FAERS/FPD-QDE-FAERS.html (no accession number is applicable).

## References

[B1] Aman A. Akram A. Akram B. Maham M. Bokhari M. Z. Akram A. (2025). Efficacy of cardiac myosin inhibitors mavacamten and aficamten in hypertrophic cardiomyopathy: a systematic review and meta-analysis of randomised controlled trials. Open Heart 12, e003215. 10.1136/openhrt-2025-003215 39988344 PMC11848667

[B2] Brown E. G. Wood L. Wood S. (1999). The medical dictionary for regulatory activities (MedDRA). Drug Safety 20, 109–117. 10.2165/00002018-199920020-00002 10082069

[B3] Chiang M. Sychterz C. Perera V. Merali S. Palmisano M. Templeton I. E. (2023). Physiologically based pharmacokinetic modeling and simulation of mavacamten exposure with drug-drug interactions from CYP inducers and inhibitors by CYP2C19 phenotype. Clin. Pharmacology Therapeutics 114, 922–932. 10.1002/cpt.3005 37467157

[B4] Cutroneo P. M. Sartori D. Tuccori M. Crisafulli S. Battini V. Carnovale C. (2023). Conducting and interpreting disproportionality analyses derived from spontaneous reporting systems. Front. Drug Safety Regulation 3, 1323057. 10.3389/fdsfr.2023.1323057 40980108 PMC12443087

[B5] Desai M. Y. Okushi Y. Gaballa A. Wang Q. Geske J. B. Owens A. T. (2024). Serial changes in ventricular strain in symptomatic obstructive hypertrophic cardiomyopathy treated with mavacamten: insights from the VALOR-HCM trial. Circ. Cardiovasc. Imaging 17, e017185. 10.1161/circimaging.124.017185 39221824 PMC11410149

[B6] Desai M. Y. Seto D. Cheung M. Afsari S. Patel N. Bastien A. (2025). Mavacamten: Real-World experience from 22 months of the risk evaluation and mitigation strategy (REMS) Program. Circ. Heart Failure 18, e012441. 10.1161/CIRCHEARTFAILURE.124.012441 39523955 PMC11745710

[B7] DeVries J. H. Irs A. Hillege H. L. (2023). The European medicines agency assessment of mavacamten as treatment of symptomatic obstructive hypertrophic cardiomyopathy in adult patients. Eur. Heart Journal 44, 3492–3494. 10.1093/eurheartj/ehad429 PMC10542624 37482673

[B8] F.A.E.R.S. (FAERS) (2023). FDA Adverse Event Reporting System (FAERS) Public Dashboard.

[B9] Garcia-Pavia P. Bilen O. Burroughs M. Costabel J. P. de Barros Correia E. Dybro A. M. (2025). Aficamten vs metoprolol for obstructive hypertrophic cardiomyopathy: MAPLE-HCM rationale, Study design, and baseline characteristics. JACC. Heart Failure 13, 346–357. 10.1016/j.jchf.2024.11.011 39909646

[B10] Hegde S. M. Lester S. J. Solomon S. D. Michels M. Elliott P. M. Nagueh S. F. (2021). Effect of mavacamten on echocardiographic features in symptomatic patients with obstructive hypertrophic cardiomyopathy. J. Am. Coll. Cardiol. 78, 2518–2532. 10.1016/j.jacc.2021.09.1381 34915982

[B11] Kalilani L. Atashili J. (2006). Measuring additive interaction using odds ratios. Epidemiologic Perspectives & Innovations EP+I 3, 5. 10.1186/1742-5573-3-5 16620385 PMC1475799

[B12] Mar P. L. Horbal P. Chung M. K. Dukes J. W. Ezekowitz M. Lakkireddy D. (2022). Drug interactions affecting antiarrhythmic drug use. Circulation. Arrhythmia Electrophysiology 15, e007955. 10.1161/CIRCEP.121.007955 35491871

[B13] Merali S. Bunn H. Sychterz C. Chiang M. Puli S. Sehnert A. J. (2025). Exposure-Response modeling and simulation to identify optimal mavacamten posology when coadministered with CYP3A4 and CYP2C19 inhibitors in patients with obstructive HCM. J. Clinical Pharmacology 65, 1802–1814. 10.1002/jcph.70072 PMC12649289 40796530

[B14] Nag S. Gollapudi S. K. Del Rio C. L. Spudich J. A. McDowell R. (2023). Mavacamten, a precision medicine for hypertrophic cardiomyopathy: from a motor protein to patients. Sci. Advances 9, eabo7622. 10.1126/sciadv.abo7622 37506209 PMC12494088

[B15] Noguchi Y. Aoyama K. Kubo S. Tachi T. Teramachi H. (2020). Improved detection criteria for detecting drug-drug interaction signals using the proportional reporting ratio. Pharm. (Basel) 14, 4. 10.3390/ph14010004 PMC7822185 33374503

[B16] Ogilvie B. W. Yerino P. Kazmi F. Buckley D. B. Rostami-Hodjegan A. Paris B. L. (2011). The proton pump inhibitor, omeprazole, but not lansoprazole or pantoprazole, is a metabolism-dependent inhibitor of CYP2C19: implications for coadministration with clopidogrel. Drug Metabolism Disposition The Biological Fate Chemicals 39, 2020–2033. 10.1124/dmd.111.041293 21795468

[B17] Ostrominski J. W. Guo R. Elliott P. M. Ho C. Y. (2023). Cardiac myosin inhibitors for managing obstructive hypertrophic cardiomyopathy: JACC: heart failure state-of-the-art review. JACC. Heart Failure 11, 735–748. 10.1016/j.jchf.2023.04.018 37407153

[B18] Perera V. Gretler D. D. Seroogy J. D. Chiang M. Palmisano M. Florea V. (2023). Effects of omeprazole and verapamil on the pharmacokinetics, safety, and tolerability of mavacamten: two drug-drug interaction studies in healthy participants. Clin. Pharmacology Drug Development 12, 1241–1251. 10.1002/cpdd.1332 37771180

[B19] Philipson D. J. Rader F. Siegel R. J. (2021). Risk factors for atrial fibrillation in hypertrophic cardiomyopathy. Eur. Journal Preventive Cardiology 28, 658–665. 10.1177/2047487319828474 30727760

[B20] Potter E. Reyes M. Naples J. Dal Pan G. (2025). FDA adverse event reporting system (FAERS) essentials: a guide to understanding, applying, and interpreting adverse event data reported to FAERS. Clin. Pharmacology Therapeutics 118, 567–582. 10.1002/cpt.3701 PMC12393772 40384638

[B21] Quarta G. Cattaneo R. S. Bonacchi G. Manca A. R. Cerillo A. G. Iacovoni A. (2026). The new life of myectomy in the era of myosin inhibitors. Prog. Cardiovascular Diseases. 95, 129–138. 10.1016/j.pcad.2026.01.004 41580106

[B22] Rader F. Oręziak A. Choudhury L. Saberi S. Fermin D. Wheeler M. T. (2024). Mavacamten treatment for symptomatic obstructive hypertrophic cardiomyopathy: interim results from the MAVA-LTE study, EXPLORER-LTE cohort. JACC. Heart Failure 12, 164–177. 10.1016/j.jchf.2023.09.028 38176782

[B23] Reyes K. R. L. Bilgili G. Rader F. (2022). Mavacamten: a first-in-class oral modulator of Cardiac Myosin for the treatment of symptomatic hypertrophic obstructive cardiomyopathy. Heart International 16, 91–98. 10.17925/HI.2022.16.2.91 36741099 PMC9872784

[B24] Reza N. Dubey A. Mahmud N. Austin M. A. Duqueney E. Patel P. (2025). Long-Term real-world outcomes of mavacamten in symptomatic obstructive hypertrophic cardiomyopathy up to 108 weeks. J. Clinical Medicine 14, 8181. 10.3390/jcm14228181 PMC12653594 41303215

[B25] Ricci F. Molinari L. V. Mansour D. Galanti K. Vagnarelli F. Renda G. (2025). Managing drug-drug interactions with mavacamten: a focus on combined use of antiarrhythmic drugs and anticoagulants. Heart Rhythm. 22, 510–525. 10.1016/j.hrthm.2024.11.041 39613202

[B26] Rothman K. J. Lanes S. Sacks S. T. (2004). The reporting odds ratio and its advantages over the proportional reporting ratio. Pharmacoepidemiol. Drug Safety 13, 519–523. 10.1002/pds.1001 15317031

[B27] Tada K. Maruo K. Isogawa N. Yamaguchi Y. Gosho M. (2020). Borrowing external information to improve Bayesian confidence propagation neural network. Eur. Journal Clinical Pharmacology 76, 1311–1319. 10.1007/s00228-020-02909-w 32488331

[B28] Wang A. Spertus J. A. Wojdyla D. M. Abraham T. P. Nilles E. K. Owens A. T. (2024). Mavacamten for obstructive hypertrophic cardiomyopathy with or without hypertension: Post-Hoc analysis of the EXPLORER-HCM trial. JACC. Heart Failure 12, 567–579. 10.1016/j.jchf.2023.07.030 37855754

[B29] Yukselen Z. Raju A. K. V. Kumar P. A. Ujjawal A. Dasari M. Parajuli S. (2024). A real-world pharmacovigilance Study of FDA Adverse Event Reporting System (FAERS) for mavacamten. Am. Journal Cardiovascular Drugs Drugs, Devices, Other Interventions 24, 791–799. 10.1007/s40256-024-00672-2 39164512

