# Supplementary material for: Heart failure reporting signals associated with concomitant mavacamten and CYP inhibitors in obstructive hypertrophic cardiomyopathy: a real-world pharmacovigilance study with propensity score matching
Source: Front Pharmacol. 2026 Jul 21;17:1846852. doi: 10.3389/fphar.2026.1846852 (PMC13433853; doi:10.3389/fphar.2026.1846852)
Supplement: Supplementary file 1 [file Supplementaryfile1.pdf]

Supplementary Table S1 Prespecified MedDRA Preferred Terms Used to Define Heart Failure-Related Events in the Primary Analysis

Outcome category	MedDRA Preferred Term	Clinical interpretation
Heart failure-related event	CARDIAC FAILURE	Clinically reported heart failure
Heart failure-related event	CARDIAC FAILURE CONGESTIVE	Congestive heart failure
Heart failure-related event	CARDIAC FAILURE ACUTE	Acute heart failure
Heart failure-related event	EJECTION FRACTION DECREASED	Reduced ejection fraction
Heart failure-related event	SYSTOLIC DYSFUNCTION	Systolic dysfunction
Heart failure-related event	LEFT VENTRICULAR DYSFUNCTION	Left ventricular systolic dysfunction
Heart failure-related event	ACUTE PULMONARY OEDEMA	Pulmonary congestion/oedema

MedDRA = Medical Dictionary for Regulatory Activities; PT = Preferred Term. These seven prespecified PTs were used in the primary analysis to capture clinically reported heart failure, systolic dysfunction, reduced ejection fraction, and pulmonary congestion events potentially related to excessive negative inotropy during mavacamten therapy.

Supplementary Table S2 Distribution of Prespecified CYP Inhibitors and Heart Failure-Related Events Among Mavacamten-Treated HOCM Reports

CYP inhibitor	Primary CYP pathway	Number of reports (n)	Heart failure-related events (n)	Event rate (%)
Omeprazole	CYP2C19	69	3	4.3
Esomeprazole	CYP2C19	21	4	19.0
Fluconazole	CYP2C19	2	0	0.0
Fluvoxamine	CYP2C19	0	0	—
Itraconazole	CYP3A4	0	0	—
Ketoconazole	CYP3A4	10	1	10.0
Clarithromycin	CYP3A4	23	3	13.0
Diltiazem	CYP3A4	60	7	11.7
Verapamil	CYP3A4	114	22	19.3
Amiodarone	CYP3A4	67	16	23.9

Heart failure-related events were defined using the seven prespecified MedDRA Preferred Terms included in the primary analysis. Because individual reports may contain more than one CYP inhibitor, the sum of drug-specific report counts may exceed the total number of reports in the combination therapy cohort. Abbreviations: CYP, cytochrome P450; HOCM, obstructive hypertrophic cardiomyopathy; MedDRA, Medical Dictionary for Regulatory Activities.

Supplementary Table S3 Heart Failure-Related Events According to Single versus Multiple CYP Inhibitor Exposure

Exposure Pattern	Number of Reports	HF-Related Events	Event rate (%)
Single CYP inhibitor	290	42	14.5
Multiple CYP inhibitors	31	5	16.1

Multiple CYP inhibitor exposure was defined as the concurrent reporting of more than one prespecified CYP2C19 or CYP3A4 inhibitor within the same FAERS report.

Supplementary Table S4 Complete-Case Sensitivity Analysis Using Continuous Age and Body Weight

Variable	Odds ratio	95% confidence interval	P value
concomitant CYP inhibitor use	3.13	1.48–6.62	0.00285
age_years	1.02	0.99–1.06	0.160
weight_kg	1.00	0.99–1.01	0.837
sexM	1.44	0.64–3.22	0.378

This complete-case sensitivity analysis modeled age and body weight as continuous variables. Heart failure-related events were defined using the seven prespecified MedDRA Preferred Terms included in the primary analysis. Because body weight was missing in a substantial proportion of FAERS reports, this analysis was considered sensitivity analysis only and did not replace the primary model.

Supplementary Table S5 Multivariable logistic regression of concomitant CYP inhibitor use in the propensity score-matched sensitivity cohort

**Variable**	**aOR (95% CI)**	***P* value**
Concomitant CYP Inhibitor Use (PSM Sensitivity Cohort)	1.99 (1.37-2.88)	< 0.001

Note: aOR, adjusted odds ratio; PSM, propensity score matching.
